# AMPKα1: A glucose sensor that controls CD8 T-cell memory

**DOI:** 10.1002/eji.201243008

**Published:** 2013-02-13

**Authors:** Julia Rolf, Marouan Zarrouk, David K Finlay, Marc Foretz, Benoit Viollet, Doreen A Cantrell

**Affiliations:** 1 Division of Cell Signalling and Immunology, University of DundeeDundee, Scotland, UK; 2 School of Biochemistry and Immunology, Trinity Biomedical Sciences Institute, Trinity CollegeDublin, Ireland; 3 Inserm, U1016, Institut Cochin, Cnrs, UMR8104, Université Paris DescartesSorbonne Paris Cité, France

**Keywords:** Cytotoxic T lymphocyte, Energy stress, *Listeria monocytogenes*, Memory, Metabolism

## Abstract

The adenosine monophosphate-activated protein kinase (AMPK) is activated by antigen receptor signals and energy stress in T cells. In many cell types, AMPK can maintain energy homeostasis and can enforce quiescence to limit energy demands. We consequently evaluated the importance of AMPK for controlling the transition of metabolically active effector CD8 T lymphocytes to the metabolically quiescent catabolic memory T cells during the contraction phase of the immune response. We show that AMPKα1 activates rapidly in response to the metabolic stress caused by glucose deprivation of CD8 cytotoxic T lymphocytes (CTLs). Moreover, AMPKα1 restrains mammalian target of rapamycin complex 1 activity under conditions of glucose stress. AMPKα1 activity is dispensable for proliferation and differentiation of CTLs. However, AMPKα1 is required for in vivo survival of CTLs following withdrawal of immune stimulation. AMPKα1^null^ T cells also show a striking defect in their ability to generate memory CD8 T-cell responses during *Listeria monocytogenes* infection. These results show that AMPKα1 monitors energy stress in CTLs and controls CD8 T-cell memory.

## Introduction

T lymphocytes respond to antigen by proliferating and differentiating to effector cells that mediate adaptive immune responses. Naïve and memory T lymphocytes are metabolically quiescent and have low rates of amino acid uptake and protein synthesis. They also have low rates of glucose uptake and use oxidative phosphorylation to efficiently metabolize glucose to generate ATP 1. However, effector T cells metabolically reprogram and upregulate glucose, amino acid, and iron uptake to support the synthesis of the new macromolecules necessary for T-cell clonal expansion and effector function. Immune-activated T cells also switch from metabolizing glucose primarily through oxidative phosphorylation to using the glycolytic pathway 2–4. These changes in metabolism appear to be rate limiting for the differentiation of both naïve CD4 and CD8 T cells into effector and memory subtypes 5–8. Accordingly, it is important to understand the mechanisms that allow T cells to increase their metabolism to meet the energy demands of effector cells. It is equally important to identify signals that allow effector T cells to return to a metabolically quiescent state as they make the transition from effector cells to memory T cells during the contraction phase of the immune response. In this context, recent studies have shown that the mammalian target of rapamycin complex 1 (mTORC1) controls the production of memory T cells 9. Inhibition of mTORC1 with rapamycin thus increases the memory T-cell formation following virus challenge. There is an increasing awareness that the control of T-cell metabolism has the potential to dictate T-cell fate.

One evolutionarily conserved molecule that controls cell metabolism is the serine/threonine kinase adenosine monophosphate-activated protein kinase (AMPK). AMPK is phosphorylated and activated by liver kinase B1 when cells are energy stressed 10. Triggering of TCR also activates AMPK via Ca^2+^–calmodulin-dependent protein kinase kinases 11. The proposed role for active AMPK is to restore energy balance in a cell by inhibiting ATP-consuming processes and stimulating ATP-generating pathways 10. In energy stressed cells, AMPK thus enforces quiescence to limit energy demands. T cells exclusively express the AMPKα1 catalytic isoform. In this context, germ-line deletion of AMPKα1 on a C57BL/6 background results in embryonic lethality (http://www.emmanet.org/mutant_types.php?keyword=0417). However, mixed genetic background AMPKα1 null mice are viable and appear fully immune-competent in vivo 12, 13. These data indicate that AMPK is dispensable for T-cell effector function. However, a recent study has shown that metformin, a pharmacological activator of AMPK promotes the production of memory T cells 14. The caveat is that metformin indirectly activates AMPK because it inhibits respiratory chain complex I and thereby causes an increase in cellular adenosine monophosphate/ATP ratio. Metformin has many effects on cell metabolism that are not mediated by AMPK 15, 16. Moreover, even if activation of AMPK can promote the formation of memory T cells, this does not inform whether AMPK is essential for this key process. Metformin actions in vivo can thus be independent of AMPK or indeed could be T-cell extrinsic 15, 16. Hence for a detailed analysis of the role of AMPKα1 in T cells, there is a requirement to examine the consequences of a T-cell-specific deletion of this kinase. Accordingly, the present study uses a CD4Cre transgene model to delete AMPKαl floxed alleles in thymocytes. We show that AMPKα1 activity is not required for CD8 T-cell proliferation or differentiation. However, AMPKα1 is shown to act as a critical sensor of energy status to control mTORC1 activity in CTLs and is required for CD8 T-cell memory.

## Results

### AMPKα1 is dispensable for proliferation, generation and function of CTLs

**Figure 1:**
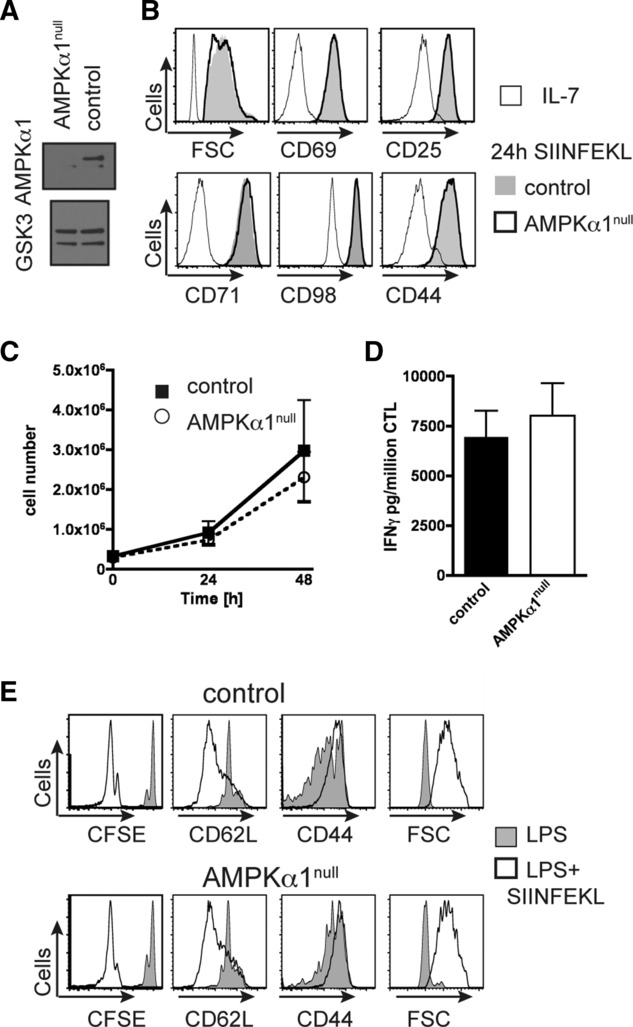
AMPKα1^null^ T cells activate, proliferate, and function normally. (A) Immunoblot analysis of total AMPKα1 and GSK3 in control and AMPKα1^null^ CD4 thymocytes, two experiments. (B) FSC, CD69, CD25, CD71, CD98, and CD44 expression by control (gray shade) and AMPKα1^null^ (thick line) OT1 LN cells activated in vitro for 24 h with SIINFEKL compared with IL-7 (thin line). (C) IL-2 maintained proliferation in vitro, control (filled squares) and AMPKα1^null^ (open circles) of OT1 cytotoxic T lymphocytes (CTLs), average ± SD, three experiments. (D) IFN-γ secretion (pg/million CTLs) 3 h SIINFEKL restimulation of control and AMPKα1^null^ OT1 CTLs. Data are shown as mean + SEM of *n* = 3 mice/genotype representing three experiments. (E) CFSE profile, CD62L, CD44, and FSC analysis of control (top panel) and AMPKα1^null^ (bottom panel) OT1 cells adoptively cotransferred into Ly5.1 recipient mice, 2 days after immunization with LPS + SIINFEKL (open) or LPS (gray shade), two experiments, two to three recipients.

**Figure 2:**
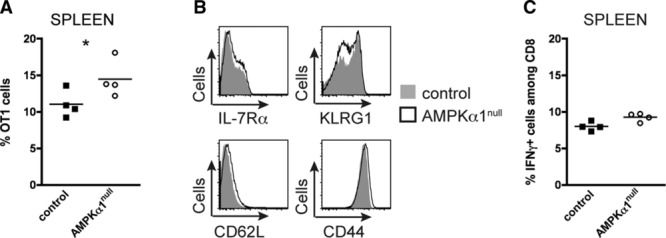
AMPKα1 is dispensable for generation of CD8 effector T cells during recombinant *Listeria monocytogenes* OVA infection. Analysis day 7 after primary recombinant *L*. *monocytogenes* OVA infection showing (A) frequency transferred control and AMPKα1^null^ OT1 cells from recipient spleens. (B) IL-7Rα, KLRG1, CD62L, and CD44 expression by transferred OT1 cells. (C) Frequency of ex vivo splenic IFN-γ-producing control and AMPKα1^null^ OT1 cells, 5 h SIINFEKL restimulation. Each symbol represents congenically marked (A) OT1 and (C) IFN-γ-producing cells among total CD8 cells from an individual recipient. (A–C) Data shown are representative of one out of two independent experiments (*n* = 4–7 recipients/experiment), paired *t*-test.

Mice with floxed AMPKα1 alleles were backcrossed to transgenic mice expressing Cre recombinase under the control of the CD4 promoter (CD4Cre) to delete the AMPKα1 gene in CD4/CD8 double positive thymocytes and hence in all subsequent T-cell populations (Fig. 1A). AMPKα1^fl/fl^ (control) and CD4creAMPKα1^fl/fl^ (AMPKα1^null^) mice had a normal distribution of peripheral α/β T cells in the thymus, lymph nodes, and spleen (data not shown). TCR primed CD8 T cells cultured in IL-2 clonally expand and differentiate to cytotoxic T lymphocytes (CTLs) 17. AMPKα1^null^ CD8 T cells activated normally in vitro (Fig. 1B), and showed normal growth and proliferative responses (Fig. 1C) and differentiated to effector cells as judged by their ability to produce high levels of IFN-γ (Fig. 1D). Further experiments examined the impact of AMPKα1 deletion on the ability of OT1 TCR transgenic CD8 T cells to activate and proliferate in vivo. Adoptively transferred OT1 cells were TCR triggered with SIINFEKL peptide presented by the MHC class I molecule H-2K^b^ by APCs. AMPKα1^null^ OT1 cells responded normally to TCR triggering in vivo and blasted and proliferated (Fig. 1E). We also determined the ability of AMPKα1^null^ OT1 cells to differentiate into effector CD8 T cells during an immune response against the attenuated bacterial strain *Listeria monocytogenes* engineered to express the OVA-derived peptide SIINFEKL, i.e. recombinant *L. monocytogenes* OVA (rLMOVA) 18. Equal numbers of control and AMPKα1^null^ OT1 T cells were adoptively transferred into recipient mice that were challenged with rLMOVA. The frequency of OT1 cells in the pathogen-challenged animals was analyzed at day 7, the peak of the effector phase. At this time point, the relative frequency of AMPKα1^null^ OT1 T cells in the spleen was modestly increased compared with control cells (Fig. 2A). Both control and AMPKα1^null^ OT1 cells had downregulated expression of IL-7Rα and CD62L and upregulated expression of CD44 and KLRG1: a cell surface phenotype characteristic of effector CD8 T cells (Fig. 2B). Control and AMPKα1^null^ cells were equally able to respond rapidly ex vivo to produce high levels of IFN-γ upon cognate antigen rechallenge (Fig. 2C). Collectively, these data reveal that AMPKα1 is dispensable for CD8 T-cell differentiation into effector cells during an immune response.

### AMPKα1 acts as a sensor of glucose metabolism in CTLs

**Figure 3:**
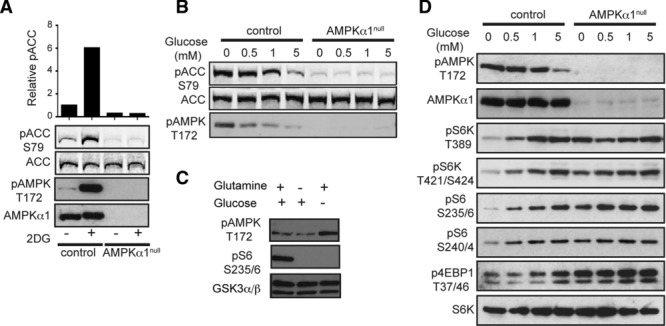
Energy stress activates AMPKα1 and inhibits mTORC1 in an AMPK-dependent manner in cytotoxic T lymphocytes (CTLs). Immunoblot detection of (A) pAMPK^T172^ and pACC^S79^ in control and AMPKα1^null^ polyclonal CTLs incubated ± 50 mM 2-deoxyglucose for 20 min. Relative pACC quantified as integrated intensity measured by the LICOR. (B) pAMPK^T172^ and pACC1^S79^ in control and AMPKα1^null^ polyclonal CTLs cultured in 0–5 mM glucose for 1 h. (C) OT1 CTLs pAMPK^T172^, pS6^S235/6^, and total GSK3-α/β after *L*-glutamine deprivation or glucose deprivation for 2 h. (D) pAMPK^T172^, total AMPKα1, pS6K^T389^, pS6K^T421/S424^, pS6^S235/6^, pS6^S240/4^, p4EBP1^T37/46^ and total S6K detection in control and AMPKα1^null^ CTLs cultured in 0–5 mM glucose for 1 h. Representative data from three experiments (A and B) and two experiments (C and D) are shown.

Effector CD8 T cells are highly glycolytic and maintain high levels of glucose uptake 19. CTLs treated with 2-deoxyglucose, an inhibitor of glycolysis, activated AMPK as judged by high levels of AMPK^T172^ phosphorylation and also increased levels of acetyl–CoA carboxylase phosphorylated on its AMPK substrate sequence Ser79 (pACC^S79^) (Fig. 3A). There was no detectable ACC phosphorylation in AMPKα1^null^ CTLs treated with 2-deoxyglucose (Fig. 3A). CTLs thus exclusively expressed the AMPKα1 catalytic subunit and do not compensate for AMPKα1 deletion by expressing AMPKα2. Glucose deprivation also activated AMPKα1; even a brief 1 h switch of T cells into low glucose (1 mM) resulted in pAMPK^T172^ stabilization (Fig. 3B). Moreover, the titratable effect of different levels of exogenous glucose on AMPKα1 activity in CTLs demonstrated the ability of AMPKα1 to act as a quantitative sensor of glucose uptake in CTLs (Fig. 3B). Recent studies have revealed the importance of energy-generating glutaminolysis pathways in T cells 8. However, glutamine deprivation did not cause AMPKα1 activation in T cells, indicating that AMPKα1 selectively monitors glucose metabolism (Fig. 3C).

One proposed function of AMPKα1 is to switch cells to a quiescent catabolic state. In this context, one conserved mechanism used by AMPKα1 to restore energy balance in cells is inhibition of mTORC1 20, 21. Previous studies have shown that glucose deprivation inhibits mTORC1 in T cells 22 but whether this is mediated by AMPKα1 has not been explored. The present experiments address this issue by monitoring the impact of glucose deprivation on mTORC1 activity in control and AMPKα1^null^ CTLs. In these experiments, mTORC1 activity was monitored by assessing the phosphorylation of mTORC1 substrate sequences in p70 S6-Kinase 1 (S6K1^T389, T421/S424^) and 4EBP-1^T37/46^. Phosphorylation of S6K substrate sequences in the S6 ribosomal subunit (pS6^S235/6, S240/4^) was also monitored. Figure 3D shows that in control CTLs, the activity of mTORC1 was strictly dependent on cells sustaining high levels of glucose uptake as even a switch into 1 mM glucose inactivated mTORC1. Strikingly, glucose-deprived AMPKα1^null^ CTLs maintained high levels of mTORC1 activity (Fig. 3D). AMPKα1 is thus a dynamic sensor for glucose uptake and functions to terminate mTORC1 activity under conditions of energy stress in CTLs.

**Figure 4:**
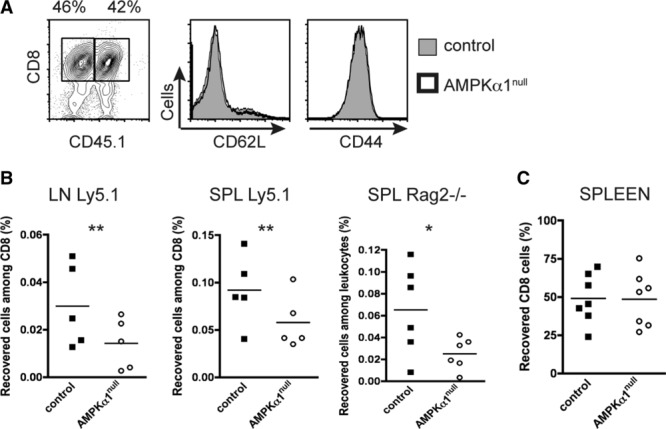
AMPK is required for cytotoxic T lymphocytes (CTLs) adaptation to metabolic stress in vivo but dispensable for lymphopenia-induced proliferation. (A) Control (gray shade) and AMPKα1^null^ (thick line) polyclonal CTLs used for adoptive cotransfer gated on CD45.1 congenic marker, expression of CD62L and CD44. (B) Frequency of cotransferred CFSE^hi^ CD8 CD45 congenically marked CTLs 7 days after adoptive transfer. Each symbol represents recovered cells from an individual Ly5.1 recipient among CD8 cells, LN left panel, spleen middle panel or Rag2^-/-^ recipient spleen among leukocytes, right panel. Data shown are pooled data from three experiments, paired *t*-test. (C) Frequency of recovered control and AMPKα1^null^ naïve CD8 T cells co-injected into Rag2^-/-^ mice at day 14 after adoptive transfer. Data shown are pooled from two experiments, each symbol represents recovered CD8 cells from an individual recipient.

These data raise the possibility that AMPKα1 might have a role in supporting the switch of effector T cells to a quiescent catabolic state. Accordingly, to explore the capacity of AMPKα1 to modulate metabolic adaptation, fully differentiated control and AMPKα1^null^ CTLs generated in vitro were mixed at a 1:1 ratio and adoptively cotransferred into naïve recipient mice (Fig. 4A). In these experiments, the majority of CTLs will die, because there is no antigen stimulation and no pro-inflammatory cytokines to sustain the expression of glucose and amino acid transporters that mediate the essential nutrient uptake required for CTLs metabolism. However, any cell that can make the switch to a metabolically quiescent state will be able to survive. The data show that 1 week after the adoptive transfer, a small subpopulation of the transferred CTLs could be recovered from secondary lymphoid organs. However, few transferred AMPKα1^null^ cells were recovered from secondary lymphoid organs compared with control cells (Fig. 4B). These results argue that AMPKα1^null^ effector T cells were impaired in their ability to make the transition back to quiescence compared with control cells.

It was important to consider whether there was a general inability of AMPKα1^null^ T cells to compete in vivo for secondary lymphoid tissue niches. We examined the ability of AMPKα1^null^ primary T cells to undergo lymphopenia-induced proliferation in competition with control T cells. In these experiments, control and AMPKα1^null^ T cells were adoptively transferred at a 1:1 ratio into Rag2^-/-^ mice. After 14 days, the recovery of control and AMPKα1^null^ cells from the recipient spleens showed that AMPKα1^null^ T cells were equally efficient at undergoing proliferative expansion in a lymphopenic environment. There was no intrinsic problem with the ability of naïve AMPKα1^null^ CD8 T cells to compete with control T cells for the homeostatic antigen and cytokine signals (Fig. 4C). The inability to recover AMPKα1^null^ effector cells after adoptive transfer reflects that effector CD8 T cells need AMPKα1 to revert to a quiescent state.

### CD8 T-cell memory immune responses depend on AMPKα1

**Figure 5:**
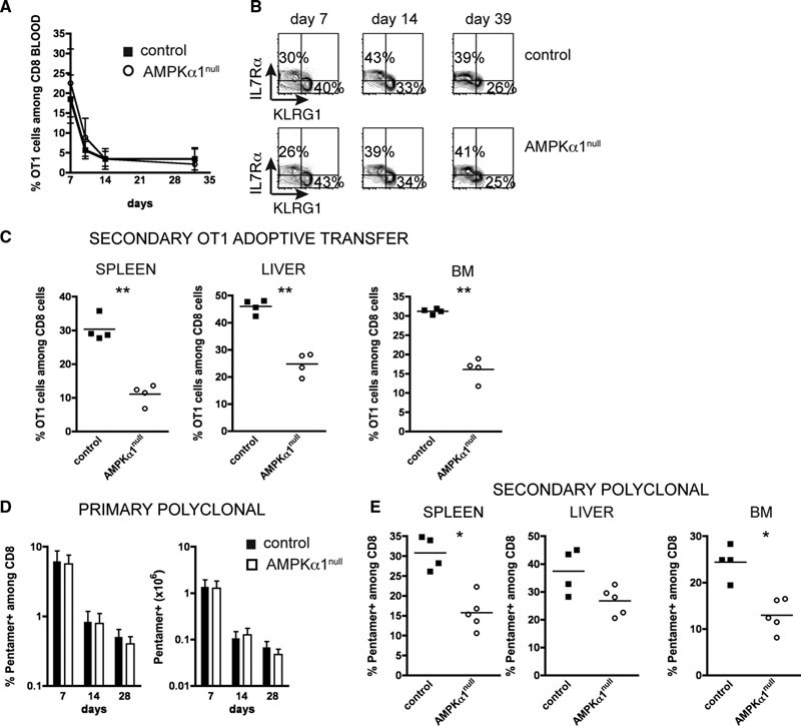
AMPK is dispensable for CD8 cells during primary infection but essential during secondary infection. (A) Frequency OT1 cells among total CD8 cells, average ± SD, eight Ly5.1 recipients, control (filled squares), and AMPKα1^null^ (open circles) in blood day 7, 10, 14, and 35 after recombinant *Listeria monocytogenes* OVA (rLMOVA) infection. (B) IL-7Rα and KLRG1 expression by OT1 cells in the blood, control (upper panel) and AMPKα1^null^ (lower panel) day 7, 14, and 39 after primary rLMOVA infection. Data shown are representative from two experiments. (C) Frequency of cotransferred control and AMPKα1^null^ TCR transgenic OT1 cells day 6 post secondary challenge in the spleen, liver, and bone marrow. Each symbol represents CD45 congenically marked OT1 cells among total CD8 cells in individual recipients. Data shown are representative from two experiments, (*n* = 4–6 recipients), paired *t*-test. (D) Frequency (left panel) and absolute number (right panel) of MHC class H-2K^b^+SIINFEKL pentamer+ cells among total CD8 cells in the spleen. Data are shown as mean ± SD (*n* = 3–5 mice/genotype/time-point). Control AMPKα1^fl/fl^ mice (black) and AMPKα1^null^ CD4creAMPKα1^fl/fl^ (open) mice days 7, 14, and 28 after primary rLMOVA infection. (E) Frequency of pentamer+ cells among CD8 cells day 6 post secondary rLMOVA challenge in control AMPKα1^fl/fl^ mice (black square) compared with AMPKα1^null^ CD4creAMPKα1^fl/fl^ mice (open circle) in spleen, liver, and bone marrow. Each symbol represents one mouse, Mann–Whitney test.

In a response to infection with rLMOVA, AMPKα1^null^ CD8 OT1 T cells undergo normal clonal expansion and contraction (Fig. 2 and 5A). Different CD8 effector subsets can be defined based on their relative expression of IL-7R-α and KLRG1 23, 24. It has been shown that IL-7Rα^high^KLRG1^low^ memory precursor effector cells have a greater potential to become long-lived memory cells and contribute to secondary responses than IL-7Rα^low^KLRG1^high^ short-lived effector cells. However, the memory precursor effector cells/short-lived effector cells distribution of OT1 T cells following rLMOVA infection was independent of AMPKα1 at various time-points after primary infection (Fig. 5B). AMPKα1 thus seems dispensable for CD8 T-cell immune responses during primary infection with rLMOVA. To address whether AMPKα1 contributes to secondary CD8 responses, control and AMPKα1^null^ OT1 cells were cotransferred and recipient mice rechallenged with rLMOVA 3 weeks following primary infection and assessed for a recall response after a further 6 days. These recall experiments revealed a striking decrease in the relative frequency of AMPKα1^null^ OT1 cells compared with control OT1 cells both at the sites of *Listeria* infection: spleen and liver and in the bone marrow (Fig. 5C). Taken together, AMPKα1^null^ OT1 cells were strikingly defective in their ability to generate a secondary immune response to rLMOVA (Fig. 5C). To further confirm whether AMPKα1 may play a role in primary infection, we analyzed the frequency and absolute numbers of polyclonal ova-specific CD8 T cells defined by MHC class I+SIINFEKL pentamer flow cytometry staining. Also in a polyclonal setting, in CD4creAMPKα1^fl/fl^ mice, AMPKα1 appears dispensable for primary CD8 T-cell responses (Fig. 5D). Figure 5E shows that CD4CreAMPKα1^fl/fl^ mice undergoing a secondary challenge to rLMOVA had reduced frequencies of ova-reactive CD8 T cells in the spleen, liver, and bone marrow compared with control mice.

AMPKα1^null^ CD8 T cells are thus impaired in their ability to make effector T cells in a secondary immune response.

## Discussion

The control of T-cell metabolism seems to be important in the effector/memory transition of T cells. The cytokine IL-2 that drives effector CTLs differentiation at the expense of memory cell formation is a strong activator of mTORC1 signaling whereas IL-15, which supports memory T-cell formation, only weakly initiates mTORC1 activity 25. CTLs have high rates of glucose and amino acid uptake to meet the energy demands associated with rapid proliferation and abundant effector cytokine production. In contrast, memory T cells are relatively metabolically quiescent cells. Effector CD8 T cells would thus need to revert from a metabolically active state to a quiescent catabolic state to produce memory T cells 26. The strength of mTORC1 activation appears to be a pivotal control switch that determines effector versus memory CD8 T-cell differentiation 9, 27. Previous studies have shown that glucose deprivation inhibits mTORC1 activity in T cells 22 but the glucose sensor that mediates this response was unknown. The present article demonstrates that AMPKα1 acts as a critical glucose sensor in effector CTLs and is activated by glucose deprivation and functions to restrain mTORC1 activity under conditions of metabolic stress.

TCR triggering activates AMPKα1 11, but AMPKα1 seems dispensable for the initial antigen receptor-induced metabolic switch that accompanies effector T-cell differentiation. AMPKα1 is not required for T-cell proliferation or the induction of CD8 effector T cells in a primary immune response to *L. monocytogenes* infection. However, AMPKα1^null^ CD8 T cells are impaired in their ability to make effector T cells in a secondary immune response. The developmental processes that regulate effector T cells progression to form memory cells must include mechanisms that allow CD8 T cells to revert from the metabolically active state characteristic of effectors to the quiescent catabolic state of memory cells. The present data are consistent with a model whereby AMPKα1 regulates the metabolic adaptations that allow secondary effector cell generation. AMPKα1 thus monitors energy stress in CD8 T cells and is a key regulator of CD8 T-cell memory to secondary effector transition during an immune response to infection.

## Materials and methods

### Mouse models

AMPKα1^fl/fl^ mice were obtained from Benoit Viollet and Marc Foretz and bred to CD4cre transgenic and OT1 TCR transgenic 28 mice. Mice were maintained in the Biological Resource unit at the University of Dundee in compliance with UK Home Office Animals (Scientific Procedures) Act 1996 guidelines.

### Cell culture

CTLs were generated as described previously 29. Briefly, polyclonal CTLs were generated in vitro using 0.5 μg/mL αCD3 (2C11) and OT1 CTLs 1 ng/mL SIINFEKL stimulation for 2 days, then expanded in 20 ng/mL IL-2 for 3–5 days. Cells were grown in complete RPMI (GIBCO), 10% heat-inactivated FCS, penicillin and streptomycin, 50 μM β-mercaptoethanol and split to approximately 0.3 × 10^6^ cells/mL daily. The in vitro generated CTLs were used day 5–7 after activation. For glucose/glutamine deprivation, CTLs were incubated 1 h in glucose-free RPMI with dialyzed FCS (GIBCO) or 2 h in *L*-glutamine-free RPMI (GIBCO). Treatment with 50 mM 2-deoxyglucose (Sigma) for 20 min was used as a positive control.

### Western blot

CTLs were gently centrifuged and then lyzed at a density of 1–2 × 10^7^/mL in Tris lysis buffer containing 10 mM Tris pH 7.05, 50 mM NaCl, 30 mM Na pyrophosphate, 50 mM NaF, 5 μM ZnCl2, 10% glycerol, 0.5% Triton, 1 μM DTT, protease inhibitors (Roche), and Calyculin A phosphatase inhibitor (Calbiochem). Proteins were resolved on SDS 4–12% polyacrylamide gel, transferred to nitrocellulose membranes, blocked in 5% milk, and incubated with primary antibody and developed using HRP-coupled secondary antibodies and ECL. Antibodies to detect pAMPK^T172^, pS6KT^389^, S6K^T421/S424^, pS6^S235/6^, p4EBP1^T37/46^ and total S6K were from Cell Signalling Technologies. pACC^S79^ was detected by anti-sheep AlexaFluor 680 and Streptavidin-IRDye800 using LICOR system (Odyssey). Antibodies recognizing pACC^S79^ and AMPKα1 were kindly provided by Professor Grahame Hardie.

### Flow cytometry

Cells (1–2 × 10^6^/sample) were incubated in Fc-block (2.4G2) at 4°C in PBS with 10% complete RPMI. Fluorescent-labeled antibodies to detect: CD8-α (53−6.7), CD25 (7D4), CD44 (IM7), CD45.1 (A20), CD45.2 (104), CD62L (MEL-14), CD69 (H1.2F3), CD71 (C2), CD98 (RL388), IL-7Rα (SB/199), KLRG1 (2F1), Vα2 (B20.1), and Vβ5.1/2 (MR9–4) were obtained from BD Bioscience or eBioscience. To detect SIINFEKL-specific cells, MHC class I H-2K^b^ coupled to SIINFEKL pentamers labeled with allophycocyanin were used according to manufacturer's instructions (Proimmune).

For cytokine staining after *L. monocytogenes* infection, splenocytes were restimulated with SIINFEKL 1 μM for 5 h, Brefeldin A was added for 4 h. Intracellular cytokine staining to detect IFN-γ (XMG1.2) was performed using eBioscience reagents according to manufacturer's instructions. Flow cytometry used BD Calibur or BD LSR Fortessa and the data were processed using FlowJo softwaree (Treestar).

### ELISA

Day 7 after initial activation OT1 CTLs were restimulated with 1 μM SIINFEKL for 3 h and the supernatant was harvested. IFN-γ ELISA was done according to manufacturer's instructions (eBioscience).

### Adoptive transfer

For in vivo proliferation, CD45 congenically differently marked OT1 cells from AMPKα1^fl/fl^ (control) and CD4creAMPKα1^fl/fl^ (AMPKα1^null^) mice were purified using CD8 T-cell isolation kit (Miltenyi) according to manufacturer's instructions. The OT1 cells were mixed at an equal ratio and were labeled with 5 μM CFSE for 10 min at 37**°**C. Approximately 4 × 10^6^ OT1 cells were co-injected iv into recipient C57BL/6 Ly5.1 mice. The mice were immunized with 40 μg SIINFEKL and 25 μg LPS ip the next day. The CFSE dilution was analyzed day 2 and 4 after immunization. For adoptive cotransfer of CTLs, day 7 fully differentiated CD4creAMPKα1^fl/+^ (control) and CD4creAMPKα1^fl/fl^ (AMPKα1^null^) CTLs with different CD45 congenic markers were mixed at equal ratio, CFSE labeled and injected intravenously into C57BL/6 Ly5.1 or Rag2^-/-^ recipient mice. The CTLs recovery was analyzed 1 week later by flow cytometry. To assess lymphopenia-induced proliferation, T cells were purified using pan-T-cell kit (Miltenyi) and 2–3 × 10^6^ polyclonal CD8 T cells were mixed at an equal ratio and adoptively transferred intravenously into Rag2^-/-^ recipient mice. The recovered cells were analyzed by flow cytometry 14 days later. To exclude the possibility of CD4cre-dependent rejection, CD4creAMPKα1^fl/+^ mice were used as controls in Figure 4.

### Listeria monocytogenes infection

In total 10^4^ OT1 cells mixed at a 1:1 ratio from CD45 congenically differently marked AMPKα1^fl/fl^ (control) and CD4creAMPKα1^fl/fl^ (AMPKα1^null^) mice were coinjected ip into C57BL/6 Ly5.1 recipient mice. Alternatively, AMPKα1^fl/fl^ and CD4creAMPKα1^fl/fl^ mice were infected to determine polyclonal responses. The mice were infected with attenuated ActA-deleted OVA-expressing *L. monocytogenes* (kindly provided by Professor Hao Shen)^18^. For primary infection 1–5 × 10^6^ and for secondary challenge 10 × 10^6^ colony forming units were injected iv.

### Statistical analysis

Statistical analysis was done using Graph Pad Prism, *t*-test was performed on paired values, and Mann–Whitney nonparametric test was used for all other data. *p < 0.05, ^**^p < 0.01, ^***^p < 0.001.

## Abbreviations

AMPK: AMP-activated protein kinase
CTL: cytotoxic T lymphocyte
mTORC1: mammalian target of rapamycin complex 1
rLMOVA: recombinant *Listeria monocytogenes* OVA
